# The Effect of Peppermint Oil on Symptomatic Treatment of Pruritus in Pregnant Women 

**Published:** 2012

**Authors:** Marjan Akhavan Amjadi, Faraz Mojab, Seyedeh Bahareh Kamranpour

**Affiliations:** a *Department of Midwifery, Islamic Azad University, Rasht, Iran.*; b *Pharmaceutical Sciences Research Center (PSRC), Shahid Beheshti University of Medical Sciences, Tehran, Iran.*; c *Department of Pharmacognosy and Pharmacobiotechnology, School of Pharmacy, Shahid Beheshti University of Medical Sciences, Tehran, Iran. *

**Keywords:** Pruritus, Itch, Pregnancy, Peppermint, Menthol

## Abstract

Itching is one of the most common skin symptoms. Generalized pruritus occurs in 1-8% of pregnant women. It can create unpleasant feeling for these women especially at nights. Most pregnant women avoid using synthetic drugs because of their side effects. Peppermint is a plant which has been used as a traditional drug in Iran. It decreases skin’s temperature. This study was done to determine the effects of peppermint oil on symptomatic treatment of pruritus in pregnant women attending to Rasoul Akram Hospital in Rasht, 2011.

In this triple-blind clinical trial, 96 randomly selected subjects diagnosed with pruritus gravidarum were studied (47 cases and 49 controls). A bottle containing 60 mL of peppermint oil 0.5% in sesame oil and identical placebos were provided to be taken twice a day during 2 weeks by the case and control groups, respectively. The severity of the itch was assessed and compared before and after the study by VAS system. The results were analyzed by SPSS. Statistical methods such as descriptive analysis, independent samples’ t-test, paired samples’ t-test and Chi-square were employed.

The severity of the itch in the treated group with peppermint oil in comparison with the placebo group, showed a significant statistical difference (p = 0.003).

In accordance with the results of this study, it seems that peppermint oil can be effective in reducing the severity of Pruritus Gravidarum. More studies with larger sample sizes are required to confidently declare the mentioned results.

## Introduction

Pregnancy is a special situation for a woman’s body (1). Hormonal changes during natural pregnancy, may considerably affect the skin. Some of the skin diseases are known as unique skin disease during pregnancy, such as Cholestasis of Pruritus Gravidarum, PUPPP, Prurigo of Pregnancy, Herpes Gestationis (Pemphigoid gestationis) and Pruritic folliculitis of pregnancy (Impetigo Herpetiformis) (2). 

Pruritus Gravidarum (PG) has extensive clinical presentations but the clearest symptom is skin itching. PG begins in the late second to early late trimester of pregnancy (mean, 31 weeks) in two-third of cases. Usually the abdomen is the primary site of involvement, then PG spreads to the chest and distal of extremities (3). It often causes irritation and restlessness for pregnant women, especially at nights (4). PG has a worldwide distribution. The incidence varies greatly with reporting criteria and even with seasons. It was found to be more frequently in the winter months (3).

Pruritus occurs in 1-8% of pregnant women (4). Treatment of PG is symptomatic and directed at controlling the pruritus (3, 5). Mild attacks need only reassurance and simple mild antipruritic preparations such as comfortable baths or cooling lotions such as menthol in aqueous cream, emulsions, primrose oil, calamine lotion and *etc. *(3, 6-8). Systemic drugs include H1-receptor antagonists, H2-receptor antagonists, cholestyramine and ursodeoxycholic acid, *etc*. (9, 10).

Regarding that most women will refuse to use chemical drugs because of their side effects and paying attention to the progress of science toward herbal medicine, using some plants beside the chemical drugs can help to treat some diseases. In Iran, peppermint is used in traditional medicine as carminative, spasmolytic, anti-nausea and as a local treatment it can include cooling and mild analgesic effects (11, 12).

Peppermint (*Mentha piperita*) is from mint family. This plant contains essential oil that its major component is menthol (50-60%) (12). By cooling the skin, menthol decreases itching that is caused by histamine. The mechanism of menthol’s effect on curing itching is unknown. The researches have shown that menthol inhibits itching by activating A-delta fibers and *k*-opioid receptor (13).

It has not been reported any data about toxic effects of using peppermint during pregnancy and breast feeding (12). Although menthol has been consumed to relive pruritus for many years, there is surprising little literature assessing its efficacy (13).

As we considered, no research has done yet about the influence of peppermint oil on curing the skin itching at pregnancy and current research is done for the first time.

The present study has performed to determine the effect of peppermint oil on symptomatic treatment of PG at pregnant women attending to Rasoul Akram Hospital in Rasht at 2011. 

## Experimental

This study was a triple-blind clinical trial. The statistical society included all pregnant women that referred to prenatal clinic of Rasoul Akram Hospital who had moderate to severe degree of skin itching, due to the visual analogue scale (VAS). On the basis of pilot sampling, to determine 2.5 unit difference in score average after the treatment of itching and estimate 2.5 unit standard deviation and considering 90% power and 95% confidence level and 30% loss coefficient, 90 subjects were calculated for samples (45 subjects in each group). Samples were chosen by availability sampling. Then, a simple random sampling was used and the subjects were divided into two groups of Case (peppermint oil) and Control (placebo).

In order to prepare peppermint oil and placebo, we coordinated with Shahid Beheshti School of Pharmacy in Tehran. Drugs were prepared under the supervision of pharmacognosist of the school. At first, peppermint oil has been prepared from Zardband Company (Tehran, Zardband) in May 2010. Then, 0.5% solutions of the oil were provided on sesame oil. Sesame oil was bought from certain groceries of markets in Tehran. Present solutions were prepared at amount of 60 mL in each dark glassy bottle. Each bottle was devoted a code, but all bottles were packed similarly. Furthermore, in order to be secure of menthol effective existence in pharmaceutical samples that are made, pharmaceutical analysis has done by related specialist. While we got the licensee, we referred to prenatal clinic of above mentioned hospital, the pregnant women who were affected by moderate or severe skin itching according to VAS and their tendency to participate at this research we started studying. Then, the questionnaire containing the recorded demographic characteristics and analyzed extent of itching in comparison with visual Analogue scale (VAS) was completed by researcher. VAS is a kind of system which evaluates the severity of itching and pain and is classified in groups such as: 8-10 severe score 4-7 moderate score 1-3 mild score

*Inclusion criteria*

Participants: were second or third trimester; had pruritus just in pregnancy; had pruritus without rash or eczema; did not have any physical or psychological diseases; had moderate to severe pruritus on the base of VAS; did not have any priority of allergy.

*Exclusion criteria*

Participants: Affected by any physical or psychological diseases during the study; Used any additional itching medication (drug or non-drug) to relief the pruritus; Used the advised drugs less than 10 days; Had presentation of rash or eczema during study.

At first, 110 subjects who had inclusion criteria were put under the study. In case group, a glassy bottle of 60 mL, 0.5% peppermint oil on the base of sesame oil was given to each sample and in the control group, 60 mL of sesame oil was given in a glassy bottle with similar package and coding.

It should be mentioned that according to the kind of research (a triple-blind clinical trial) the researcher, research units and statistician weren’t aware of what kind of drugs were used by patients. They should use the drug as topical and twice a day for the area which was affected by skin itching. Two weeks later, researching units have been evaluated for the severity of skin itching by means of VAS.

At last, 96 subjects remained and 49 of them were accidentally put into placebo consumer group and 47 of them in consumer of peppermint oil group.

All data analysis was being performed in SPSS. In order to describe subject’s data, two statistic methods of descriptive and analytic were used. Descriptive methods including frequency distribution table, mean index and standard deviation were applied to describe the characteristics of people who were under the study. To compare the severity of itching between the case and control groups, the independent t-test was used and to compare the situation before and after the intervention in groups, paired t-test, and for quality comparison of severity of itching, the Qui-square test was used.

## Results

Ninety-six subjects with moderate to severe skin itching according to VAS were divided into 2 peppermint (47) and placebo (49) groups. The subjects in the 2 groups had no significant difference in terms of age, job, educational level, gestational age (at the first of study), gravid, abortion history and itching history (Table 1).

**Table 1:** Demographic characteristics of the 2 groups

**p-value**	**Peppermint (n = 47)**	**Placebo (n = 49)**	**Groups**	
			**Characteristics**	
0.126 0.144 0.150 0.786 0.367 0.445 0.196	27.82 (3.72)	25.9 (3.71)	Age (mean, SD)	Job (%) Educational Level (%) Gestational Age at the first of study (%) Gravida (%) *Abortion History (%) **Itching History (%)
	45 (95.7)	49 (100)	Housewife	
	2 (4.3)	0	Employee	
	19 (40.4)	27 (55.1)	Under Diploma	
	28 (59.6)	22 (44.9)	Diploma and higher	
	12 (26.1)	14 (28.6)	Second Trimester	
	34 (73.9)	35 (71.4)	Third Trimester	
	30 (63.8)	29 (59.2)	Primigravida	
	17 (36.2)	20 (40.8)	Multigravida	
	44 (95.7)	45 (91.8)	No	
	2 (4.3)	4 (8.2)	Yes	
	6 (35.3)	9 (50)	No	
	11 (64.7)	9 (50)	Yes	

*: multigravida subjects

In comparison of frequency distribution, the difference of the severity of itching before and after the treatment between the case and control group, the results on the basis of Qui-square test showed that the severity of itching after the treatment in both groups had meaningful statistical difference (p = 0.02) (Table 2).

**Table 2:** Comparison of itching severity according to VAS in 2 groups before and after the intervention

**p-value**	**Peppermint Frequency (%)**	**Placebo Frequency (%)**	**Groups Itching severity**	**Time**
0.74	35 (74.5)	35 (71.4)	Moderate	Before the Intervention
	12 (25.5)	14 (28.6)	Severe	
0.02	41 (87.2)	30 (61.2)	Mild	After Intervention
	4 (8.5)	12 (24.5)	Moderate	
	2 (4.3)	7 (14.3)	Severe	

According to the results, itching severity on the base of VAS in peppermint group was decreased from 5.9 to 3.25 and in the placebo group from 5.76 to 1.06. For comparing the mean of itching severity before and after the treatment in both case and control group, we used paired t-test that showed meaningful statistic difference in each group (p = 0.001) (Table 3).

**Figure 1:**
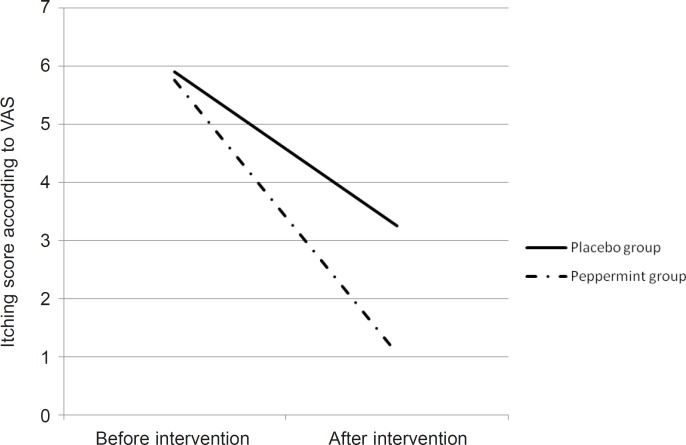
Comparison of itching scores in the 2 groups before and after the intervention

In order to compare the differences, we used independent t-test which didn’t show meaningful statistic difference about the severity of itching before the treatment between the two groups but after the treatment, the difference was statistically meaningful in both groups (p = 0.003) (Table 3 and Figure 1).

**Table 3:** Mean differences of itching severity score according to VAS in the 2 groups before and after the intervention VAS

**p-value**	**Peppermint**	**Placebo**	**Times**
0.61	5.76 (1.15)	5.9 (1.74)	Before the intervention, mean (SD)
0.003	1.06 (1.5)	3.25 (2.5)	After the intervention, mean (SD)
	0.001	0.001	p-value before and after the intervention

## Discussion

Though the skin itching at pregnancy can be created for many reasons, the usual symptomatic treatment of itching without paying attention to its creating cause would be the use of local strode and emulsions (4).

Therefore, using the drug that could decrease the severity of skin itching in those women, can bring back their peace. The results of this research showed that the severity of itching after the treatment between the two groups had a meaningful difference (p = 0.02). In comparison with the ursodeoxycholic acid versus the cholestyramine for liver cholestasis, results showed that the severity of itching had more reduction in ursodeoxycholic acid group (66.6% in contrast to 19%) (14). Therefore, it is noticeable that the peppermint oil can be effective for the treatment of itching during the pregnancy, as well as chemical drugs which have side effects. Considering that the effective material of peppermint oil for the treatment of itching is menthol and there isn’t any registered clinical trial for menthol’s effect on PG, so, the researcher decided to compare the result of research with the results of articles which considered the menthol’s effect on the other kind of skin itching.

In comparison of the influence of phenol and menthol combination (1%) for treating the lesion presentations of itchy skin, between the patients who were injured chemically by Sulfur Mustard and the patients who were injured unchemically, it was shown to improve itching in 15% of chemical patients and 20% of unchemical ones (15). It seems that on the base of these results, we will be able to use peppermint oil which contains menthol, in order to improve PG during the pregnancy.

Finally, taking into account the effective material of peppermint oil called menthol, for external use, it is a dominant component in ointment that has cooling effect and mild analgesic (12). On the base of current results, peppermint oil can be consumed for symptomatic treatment of skin itching on the pregnant women. The results of this study have shown that peppermint oil didn’t crest any special side effects on subjects. But we should consider the lack of clinical trial in this field, so, it should be advised that extensive researches in different society with numerous samples should be implemented. 

Limitations of study: Data gathering was just based on the statements of participants. The continuing of the study was given up. 

## References

[1] Barhu G (2003). Viral Infections and Skin in Pregnancy.

[2] Cunningham FG, Leveno KJ, Bloom SL, Hauth JC, Rouse DJ, Spong CY (2010). William’s Obstetrics.

[3] Ingber A (2009). Obstetric Dermatology.

[4] Peharda V, Gruber F, Kaštelan M, Brajac I, Čabrijan L (2000). Pruritus an important symptom of internal diseases. Dermatovenerologica.

[5] Jones DK, Weiner CP, Steer PI, Gonik B (2005). High Risk Pregnancy.

[6] Fraser DM, Cooper A, Nolte AGW (2008). Myles Textbook for Midwives.

[7] Jones SV, Black M (1999). Pregnancy dermatomes. J. Am. Acad. Dermatal.

[8] Twyeross R, Greaves MW, Handworker H, Jones EA, Libretto SE, Szepietowski JC, Zyliez Z (2003). Itch: scratching more than the surface. Inter. J. Med.

[9] Dawn A, Yosipovitch G (2006). Treating itch in psoriasis. Dermatology Nursing.

[10] Warren R, Heymann MD (2006). Itch. J. Am. Acad. Dermatol.

[11] Amin Gh (2008). The Most Common Herbal Traditional Drugs in Iran.

[12] DeSmet PAGM, Keller K, Hansel R, Chandler RF (1998). Adverse Effects of Herbal Drugs.

[13] Tejesh P, Shiuji YI, Yosipovitch G (2007). Menthol: a refreshing look at this ancient compound. J. Am. Acad. Dermatol.

[14] Kondrackiene J (2005). Efficacy and safety of ursodeoxycholic acid versus cholestyramin in interahepatic cholestasis of pregnancy. Gastroenterology.

[15] Panahi Y, Davoudi SM, Khalili H, Pourheidari Gh, Bigdeli M, Taghizadeh M, Tajik A (2005). In comparison to the influence of combination of phenol and menthol (1%) for treating itchy skin lesion presentations, about patients who were injured chemically by Sulfur Mustard and unchemical paitients. Military Med. J.

